# Role of Maternal Empowerment in Addressing Child Malnutrition: Evidence from Asian Developing Countries

**DOI:** 10.3390/children12050597

**Published:** 2025-05-04

**Authors:** Mariam Abbas Soharwardi, Najma Iqbal Malik, Razia Anjum, Muhammad Sohaib Haleem, Inam Ullah Leghari, Jam Bilal Ahmad, Rohma Maryam, Maimoona Nazir, Saireen Fatima, Farooq Ahmed, Kun Tang

**Affiliations:** 1 Department of Economics, Islamia University of Bahawalpur, Bahawalpur 63100, Pakistan; 2 Department of Psychology, University of Sargodha, Sargodha 33600, Pakistan; najma.iqbal@uos.edu.pk; 3 Department of Psychology, Bath Spa University, Ras al Khaimah 71705, United Arab Emirates; razia@bathspa.ae; 4 UNICEF, Hafizabad 54782, Pakistan; 5 Department of Anthropology, Quaid-I-Azam University, Islamabad 44000, Pakistan; 6 Taxila Institute of Asian Studies, Quaid-I-Azam University, Islamabad 44000, Pakistan; jam_bil@hotmail.com; 7 Department of Biotechnology, The Islamia University of Bahawalpur, Bahawalpur 63100, Pakistan; rohmamaryam28@gmail.com; 8 Bakhtawar Amin Medical & Dental College, Multan 60000, Pakistan; 9 Fazaia Medical College, Air University, Islamabad 44000, Pakistan; 10 Department of Anthropology, The Islamia University of Bahawalpur, Bahawalpur 63100, Pakistan; 11 Vanke School of Public Health, Tsinghua University, Beijing 100084, China

**Keywords:** maternal empowerment, child malnutrition, binary logistic regression, developing Asian countries, composite maternal empowerment index (CMEI)

## Abstract

Background: Malnutrition among expectant mothers in underdeveloped areas is abundant and a serious public health concern. This study examines how maternal empowerment affects nutritional outcomes among under-five children in developing Asian nations. Objective: With an emphasis on nutritional outcomes, including stunting, wasting, and underweight, the main objective of this study is to investigate the connection between mother empowerment and child malnutrition and explore how better child health in developing Asian nations can be achieved through empowering mothers. Methods: Using Demographic and Health Survey (DHS) data of ten emerging Asian countries from three regions of Asia, this study evaluates maternal empowerment using the composite maternal empowerment index (CMEI) and examines how it relates to children’s nutritional health. For the assessment of the significance of the association between maternal empowerment and child health outcomes, statistical analysis was conducted. Results: Our results indicated that maternal empowerment and child health have a statistically significant relationship, especially regarding a reduction in the prevalence of stunting, wasting, and underweight conditions in children. At the same time, maternal education showed a significant role in reducing malnutrition in children in all three regions of Asia. Conclusion: In conclusion, developing Asian nations require empowering women. Also, it is essential to initiate nutrition programs, extension education, and synergistic working models that are especially suited to rural women. By strengthening mothers’ roles in promoting their children’s health, these initiatives can help solve the widespread problem of child malnutrition.

## 1. Introduction

Maternal empowerment is crucial for children’s nutritional consumption. Empowering mothers through awareness about nutritionally rich food is vital for reducing the chances of malnutrition in children [1,2]. Maternal diet during lactation and pregnancy directly affects the growth of a child in the first few years [3]. Because of a mother’s inadequate dietary intake, stunting frequently starts during pregnancy [4,5]. An undernourished teenage girl cannot give birth to a healthy child [6,7,8]. A woman’s health during pregnancy and a child’s health from 0 to 59 months of age play a critical role [9,10]. Of the children below the age of 5 years, 155 million are stunted, 52 million are wasted, and 17 million are severely wasted [11]. Lack of financial resources to purchase proper nutritious food for their children is one of the major reasons for increasing malnutrition in developing regions [12,13,14]. Undernutrition causes about 45% of deaths in preschool children, and most of such deaths happen in low- and middle-income countries [11]. In many developing regions, mothers lack awareness about the appropriate nutrition intake during pregnancy, which, in turn, increases the chances of malnutrition in their children [6,9,15].

The decision-making power of women is a critical factor in their empowerment. However, in some cases, even when mothers have the financial capacity, they are not able to make independent decisions regarding food. The head of the household, a mother-in-law or husband, dominates in decisions about maternal and grandchildren’s health and food in a joint family setup [16,17]. Previous literature has empirically tested the association between the health of a child and parental investment [10,16]. A study in Uganda has explored the connection between child health, fertility, and the education of women [18]. Research conducted in Bangladesh and India has found a link between stunting in children and women’s empowerment [19].

An estimated 120 million women in underdeveloped nations are underweight, and 50% of pregnant women are anemic [20]. Unlike males, females are inclined to reason and decide differently with distinct preferences, with women favoring children’s well-being and care [21]. The household’s health mostly depends on the choices of nutrition made by women. Studies indicated that when women possess knowledge and independence in decision-making, they are better equipped to select appropriate diets for their children [22]. Moreover, women are empowered because of education and skill development, which enables them to improve the social and health status of their spouses, children, and other family members [23].

Maternal empowerment and child nutritional status are closely interlinked. Factors that help a mother to be healthy and then to be better able to take care of her children include the mother’s work status, decision-making autonomy, awareness through media, self-confidence, and self-esteem. The greater the autonomy women have in decision-making toward health, buying, spending income, and visiting family and relatives, the more likely they are to take better care of their families and children, meaning better overall health for themselves [20]. The relationship between a woman’s work status and a child’s nutritional status is indeed complex and can vary significantly depending on the context. For example, there is evidence that a mother’s employment has a positive effect on child nutritional outcomes, such as in Nigeria [24], Pakistan [22], and India [7,25]. However, few studies also found a negative relationship between a mother’s work status and a child’s nutritional status [25]. Awareness of healthcare precautions has a noteworthy positive impact on a child’s nutritional status [2]. Moreover, a woman’s self-esteem plays a key role in increasing her capability for family and child care [22].

An in-depth literature review on child health and women’s empowerment revealed gaps. For example, most studies measuring child health and women’s empowerment used only one or two indicators or determinants [18]. Moreover, most studies are restricted to one or more countries, and the results cannot be generalized. Therefore, an analysis measuring linkages between the empowerment of mothers and malnutrition among children in diverse regions was required. This study addresses both major issues by measuring a comprehensive framework for maternal empowerment through five dimensions and nineteen indicators and child malnutrition through indicators of stunting, underweight, wasting, and the Composite Index of Anthropometric Failure (CIAF) across ten developing countries in Asia. Thus, this study aims to fill the literature gap and empirically test the proposed framework across regions to enhance its generalizability.

## 2. Materials and Methods

### 2.1. Theoretical/Conceptual Framework

Malnutrition was assessed using CIAF, which consisted of three malnutrition indicators: height for age (HAZ), weight for height (WHZ), and weight for age (WAZ). CIAF was utilized in this research to investigate the nutritional status of children. Grossman and Anderson’s model, as well as Newman’s model, assisted as the basis for the conceptual framework for child health, offering a reliable resource for researching health consequences [26].

**Figure 1:**
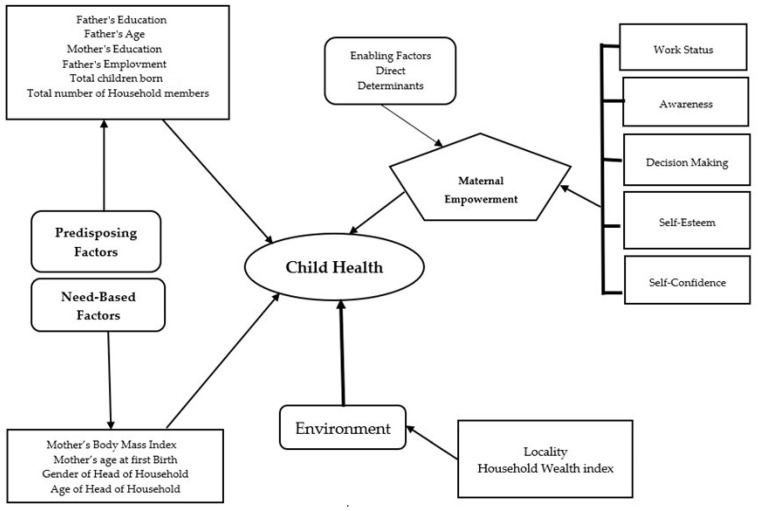
Projection of the Anderson and Newman model on child health.

Figure 1 shows the Anderson and Newman models [26] on child health, projecting the current study’s conceptual framework. Infant and children’s health may improve as a result of the interaction between enabling factors and child health. Such elements must be present for the family to take action at a reasonable cost or meet the requirement for nutrition. Enabling elements enable the family to give their children access to all the resources necessary for their health. Predisposing factors, including the mother’s education, the father’s age, and the total number of children born, were evaluated in relation to child nutrition using Anderson and Newman’s model. Improvements in child nutrition status are linked to these factors.

Need-based factors, such as how individuals perceive their general health status, their experience of pain or worries, and their encounters with diseases, are also crucial in determining child health. In the current model, a mother’s Body Mass Index [BMI] and mother’s age at first birth [MAFB] significantly influence the health of her child.

Environmental factors are equally important in shaping child health. Factors such as the place of residence, household wealth, secular states, and regions are all critical in determining the overall health outcomes for children. These environmental variables are closely correlated with child health, influencing not only the immediate health outcomes but also long-term well-being.

### 2.2. Data Collection and Set of Countries

Nationally representative DHS surveys were assessed to obtain data on the following: decision-making, women’s work status, self-confidence, awareness, self-esteem, wasting, stunting, underweight, family planning, fertility, and maternal–child health. In these surveys, the inclusion criteria for women of reproductive age were 15–49 years, and for men, 15–59. We selected 10 developing countries from 3 distinct Asian regions: Western, Central, Southern and Southeastern. Similar indicators of mother empowerment and child health were extracted from the DHS surveys across all selected countries, and the same indicators were extracted from the latest annual survey of 33 countries. The portrayal of countries chosen for analysis is given in Table 1.

### 2.3. Principal Component Analysis and Factor Analysis Used in the Construction of the Composite Maternal Empowerment Index (CMEI)

Factor analyses were used to reduce a large set of observed indicators related to maternal empowerment into a smaller number of underlying dimensions (factors) and to use these factors to create a single composite index. This approach ensures dimensionality reduction while capturing maximum variance and minimizing multicollinearity. This method was applied to identify the latent construct (dimensions of empowerment) and extract factor scores.

These factor scores represent the estimated level of each case (country) on each latent dimension. The factor scores were calculated using the regression method in SPSS 21, meaning each standardized variable was multiplied by its factor loading and summed. Five factors were retained based on the eigenvalue criterion (>1) and explained 60.45% of the total variance, which is statistically sufficient in social sciences (above the 50% benchmark).

Each factor’s contribution explained the overall variance in the data, as well as the likewise scores as a variable in subsequent calculations. The total five factors explicated 60% of the total variation, with the 1st, 2nd, 3rd, 4th, and 5th factors illumination percentages as 19.27, 12.68, 11.88, 9.31, and 7.31, respectively (Appendix A). For the CMEI, the proportion of the percentages of factors (scores) were employed as weights on the factor score coefficients through the variance-weighted factor score method. This method allows each factor to contribute proportionally to its explanatory power.

CMEI = (19.277/60.451) (score of Factor 1) + (12.681/60.451) (score of Factor 2) + (11.876/60.451) (score of Factor 3) + (9.310/60.451) (score of Factor 4) + (7.307/60.451) (score of Factor 5). (1)

As the index value might be + or − (difficult to interpret), we developed a Standardized Index (SI)—the value of which could be from 1 to 5 when we use this formula:

SI = max(CMEI) − min(CMEI)CMEI − min(CMEI) × (5−1) + 1(2)

This transformation ensures:

- Comparability across countries;
- Positive values only;
- Uniform interpretation (1 = lowest empowerment; 5 = highest).

In earlier studies [13,23], an analogous technique was used. This index, which is in the range of 1 to 5, is further divided into 3 groups [24,26] by the formula (i.e., I ≤ H-L/k) using frequency distribution cut-offs:

- Low empowerment: 1.27 to 2.36;
- Medium empowerment: 2.36 to 3.45;
- High empowerment: 3.45 to 4.

Maternal empowerment in the three regions of Asia is estimated by this single index (CMEI) ranging between 1 to 5 (lower to higher empowerment), as shown in Table 2.

According to Table 2, Nepal seems to have high rankings according to the composite MEI, which Principal Component Analysis and Factor Analysis estimate. West Asia showed a higher mean score on the maternal empowerment index. Meanwhile, Timor-Leste and Pakistan rank the lowest in terms of the mean score of the maternal empowerment index.

### 2.4. Model Specification of Empowering Mother and Child Health

We used the binary logistic regression (BLR) model in Equation (3) to apply our theoretical model to the data.

(3) $$\mathrm{Child}\;\mathrm{Malnutrition}=\beta_{0}+\beta_{1}CMEI+\beta_{2}MAFB+\beta_{3}MBMI+\beta_{4}FE+\beta_{5}GHH+\;\beta_{6}AHH+\beta_{7}ME+\beta_{8}TNCEB+\beta_{9}THM+\beta_{10}WI+\beta_{11}MA+\beta_{12}FA{+\mu }_{i}$$

We describe the variables and their measurement scales in Table A1 and the dimensions of ‘Mothers Empowerment’ and their measurement scale in Table A2 (see their details in the Appendix A).

## 3. Results

Three regions (Central Asia, West Asia, and South and Southeast Asia) were selected for separate examination. The association of maternal empowerment with children’s health was assessed using three BLR models.

### 3.1. Empowerment of Mothers and Malnutrition in Children in South and Southeast Asia

The health conditions of South and Southeast Asian children were measured based on stunting, wasting, and being underweight, as well as the CIAF indicators. The number of children, maternal and paternal characteristics, household characteristics, and mother’s empowerment are all independent characteristics; however, these have been the measures of the dependent variable factors (Table 3). Applying BLR examines the influence and direction associated with mothers’ empowerment and other relevant attributes on their child’s nutritional status.

According to the results in Table 3, all the indicators are differently affecting the malnutrition of children; they include stunting, underweight, wasting, and CIAF. The result of BLR showed that the total no. of children (ever born > 4) has a positive and strong impact on wasting, stunting, underweight, and CIAF. The model displayed that the greater the number of children born, the greater the chances of having stunting, wasting, underweight, and CIAF, which is well supported by the results of BLR.

### 3.2. Maternal Empowerment vs. Child Malnutrition in Central Asia

We selected wasting, stunting, underweight, and CIAF as the dependent variables. Using independent variables, maternal empowerment, mother and father characteristics, household characteristics, and sibling characteristics are given in Table 4. BLR is implemented to identify the relationship between a mother’s empowerment and all other traits within its path that it exerts toward a child’s malnutrition.

The CMEI was developed to measure mothers’ empowerment and subsequently find out what influence it created on children’s health. The results from BLR revealed the lack of definite influence of mother empowerment on the health conditions of their children in Central Asia, either in terms of the indicators of stunting and wasting as well as other forms of CIAF. Stunting and underweight problems in children were not significantly associated with women’s empowerment.

Four parameters of the mother’s characteristics were assessed: age at first birth, education level, BMI, and age. Stunting, wasting, underweight, and CIAF are among the child health markers that are impacted differently by each of these traits. From the results in Table 3, using the BLR model, it emerged that stunting, wasting, and underweight were not affected by a mother’s BMI being <18.5 kg/m^2^, various levels of maternal education, or the rise in the mother’s age at their first birth. However, CIAF was affected immensely due to a maternal BMI of more than 18.5 kg/m^2^. In addition, there was a positive and significant relationship between the rise in age at the first birth and wasting. Conversely, stunting, wasting, and underweight were negatively and significantly associated with the age of the mother. These results highlight the significance of maternal characteristics in influencing child health outcomes.

Two factors—the father’s age and level of education—were used to gauge his traits. Child health indicators are impacted differently by each factor. The BLR results, as displayed in Table 3, showed that while an increase in the father’s age was positively and substantially associated with stunting, wasting, and underweight, but not CIAF, the father’s level of education had no discernible effect on the health of the child. This implies that, in some areas of a child’s health, the father’s age matters more than his level of schooling.

Factors including the head’s age, gender, wealth, number of household members, and housing style were used to evaluate household characteristics. The impact of each trait on child malnutrition metrics varies. Table 5 showed that using BLR, the age of the head of household was not significant for child malnutrition, while the HH being male had a negative and significant relationship with child malnutrition. Urban residence was not significant for stunting, wasting, underweight, and CIAF. However, children with more than five household members were less likely to be stunted, wasted, or underweight. These results highlight how household variables influence the health of children.

The number ever born was used for calculating the sum of children, and individually, it has a slight effect on child malnutrition measures. All these point to the fact that the number of children has diversified impacts on various child malnutrition performance indicators.

### 3.3. Maternal Empowerment and Child Malnutrition in West Asia

Using CIAF as the response variable, markers such as stunting, wasting, and underweight were applied to assess the malnutrition status of children in West Asia. Table 5 includes independent variables, which include the mother’s empowerment, mother and paternal traits, household characteristics, and the number of children. The effects of these variables on child health and their corresponding impact directions were examined using BLR.

## 4. Discussion

In order to evaluate the effect of women’s empowerment on child health, assessments were conducted in three developing Asian regions: South and Southeast Asia, Central Asia, and West Asia. This association was measured using the composite women empowerment index. The need for a comparative analysis was highlighted by the BLR analysis results, which showed different outcomes across the areas. Our results emphasize the requirement for targeted interventions in South and Southeast Asia, where child malnutrition challenges are most pronounced, and suggest that enhancing mothers’ decision-making autonomy, access to resources, and overall empowerment could lead to significant improvements in child health outcomes [25]. Stunting and underweight are less frequent in children when their mothers are more empowered [28,29]. In essence, each of the four variables—maternal BMI, mother’s education, mother’s age, and mother’s age at first birth—confers a specific effect on the health of the child, including stunting, wasting, underweight, and CIAF. The results of BLR showed that maternal characteristics such as a BMI below 18.5 kg/m^2^ [29], all educational categories [30], and an increase in the mother’s age at first birth [31] have a detrimental and significant role in a reduction in child malnutrition, including stunting, wasting, underweight, and CIAF. On the contrary, stunting, wasting, and underweight are strongly and positively affected by advancing maternal age [15]. From the model, the results of BLR affirm the influence of maternal characteristics on the malnutrition of the child, including stunting, wasting, underweight, and CIAF. Two variables measured a father’s characteristics: his age and education level. Each factor exerts a unique effect on a child’s malnutrition, which encompasses stunting, wasting, underweight, and CIAF, consistent with the results of Table 3. The results of BLR showed that the educational background of fathers [3] and age [25] significantly and negatively influence child malnutrition through stunting, wasting, and underweight, except for CIAF. In contrast, according to the model, the outcomes of BLR are supposed to support the father’s characteristics that affect the health of the child, for instance, stunting, wasting, underweight, and CIAF [31,32].

Three distinct factors—the head’s gender, age, wealth status, household members, and locality in terms of urban and rural areas—are used to quantify the household’s characteristics. Each of these factors has a unique effect on child malnutrition, including stunting, wasting, underweight, and CIAF. According to the results of BLR, the health status of children is negatively and significantly affected by the age of the household’s head, with an increase in wealth status [33,34] and living in an urban area [35]. Among the health problems that children are more likely to experience when there are more than five household members, stunting, wasting, underweight, and CIAF are some of them [27]. The model resulting from BLR supports the effects of household factors on children’s health, such as stunting, wasting, underweight, and CIAF. Households with fewer than or equal to five members have a significant and negative impact on stunting, wasting, underweight, and CIAF.

The CMEI was used to measure maternal empowerment and ascertain how it affected children’s health [36]. The BLR results showed that maternal empowerment had negligible effects on underweight, stunting, wasting, and the CIAF in the West Asia area. This implies that the frequency of stunting and underweight among children in this area is not significantly impacted by maternal empowerment [31].

Four parameters were used to evaluate the mother’s characteristics: age at first birth, education level, BMI, and age. Every dimension has a unique impact on a child’s health metrics, such as CIAF, wasting, stunting, and being underweight. Findings from the BLR analysis indicated that different degrees of maternal education were associated with a maternal BMI below 18.5 kg/m^2^ [37]. Moreover, an increase in maternal age at the first birth had an insignificant effect on stunting, wasting, underweight, and CIAF. Conversely, a maternal BMI greater than 18.5 kg/m^2^ significantly impacted CIAF. These findings emphasize that while certain maternal characteristics may not significantly affect some child health outcomes, they still play a role in others, such as CIAF [38].

The father’s characteristics were assessed through two variables: the father’s education and the father’s age. Each factor affects child health indicators differently. The results of the BLR disclosed that stunting, wasting, and underweight were negatively and significantly impacted by the father’s age and level of education [38] but not by CIAF. This implies that while a father’s education may not directly impact CIAF, it does influence other child health outcomes significantly.

The gender, age, wealth, number of household members, and type of dwelling of the head of the household were among the parameters used to examine household characteristics. The impact of each trait on child health metrics varies. Table 4 shows the findings of BLR, which indicated that stunting, wasting, and underweight were not significantly impacted by the age or gender of the household head [male]. However, while underweight was negatively impacted, stunting and wasting were positively and considerably impacted by a higher income status [39]. Stunting and underweight were negatively and significantly impacted by living in an urban area, and children who lived with more than five household members were more likely to experience wasting.

The number of children was assessed through two variables: (1) the total number of children alive and (2) the total number of children ever born. Each variable affects the child health indicators differently. Table 4 and Table 5 reveal that having more than four children, whether alive or ever born, had a positive but insignificant effect on underweight and CIAF. However, there was a negative and significant relationship between the number of children and stunting and wasting [14,18]. These findings indicate that while having a larger number of children may not significantly impact some health outcomes, it is negatively associated with stunting and wasting. These analyses underscore the complex interplay of various factors affecting child health across different regions [40].

The results showed that in both Central and West Asia, the composite maternal empowerment score had a negligible impact. This implies that the health of children in these areas is not greatly impacted by female empowerment. The reality that women in these fields already have a fair amount of authority could be one explanation for these negligible outcomes [35,36]. They may carry out home and professional tasks with less constraint since they have the freedom to talk, make health-related decisions, show self-confidence, and enjoy respect from society [4,10].

On the other hand, the analysis demonstrated that South and Southeast Asia was significantly impacted by the composite maternal empowerment index. This suggests that child health in these areas is significantly impacted by women’s empowerment. South and Southeast Asian women still lack full empowerment; they need more autonomy, respect, trust, and decision-making authority in both their personal and professional lives. Enhancing maternal empowerment can boost their self-esteem and effectiveness by motivating and empowering them to balance power in their social, professional, and domestic lives [41]. Additionally, it is anticipated that women’s empowerment may lessen problems like underweight, wasting, and stunting in their offspring [40].

## 5. Strengths and Limitations of the Study

### Strengths

*Interdisciplinary Approach and Comprehensive CMEI Index:* By integrating interdisciplinary data, this study provided a comprehensive analysis of the factors influencing children’s health. We developed an inclusive index to assess maternal empowerment, incorporating five dimensions and 19 indicators.

*Geographic Diversity:* Additionally, this study encompassed three geographically diverse Asian regions, enabling a comparative assessment of regional variations in child health.

*Limitations:* Nevertheless, some limitations should not be ignored.

*Insufficient data:* DHS data have been used to analyze maternal empowerment’s influence on child malnutrition; in DHS, some countries’ data have not been adequately updated. Consequently, only ten Asian countries were selected for analysis with updated data after 2005, and having the data on the identical indicators employed for mothers’ empowerment and child malnutrition.

*Lack of political and cultural consideration:* We dealt with only “maternal empowerment and child malnutrition” and ignored factors of “government spending, medical facility, women’s political participation, and culture and religion”. Regional differences can also be attributed to various factors, including political conditions, environmental variations, religious influences, economic status, levels of awareness, availability of health facilities, educational attainment, family structure [joint or nuclear], opportunities for growth, use of media technologies, and government interventions [42,43,44,45,46,47,48,49]. But, due to limitations based on the DHS survey, these factors are not addressed.

*Methodological limitations:* Our results vary significantly across regions, contributing to the differing outcomes observed in BLR analyses due to these factors. This study underscores the necessity for region-specific policies and interventions to address the unique challenges and leverage the distinct opportunities for enhancing women’s empowerment and improving child health across different regions in developing Asia. For child malnutrition, only CIAF was calculated by stunting, wasting, and underweight preschool children. The study is limited to married women having children under five years of age.

## 6. Conclusions

This study suggests policy recommendations that could contribute to improving the empowerment of women and enhancing child health. First, the government should develop comprehensive educational programs tailored to different educational levels, including informal education facilitated through social media and community outreach. Designing accessible and engaging learning modules for uneducated women can significantly contribute to their empowerment and, consequently, better child health outcomes. Additionally, the government should prioritize women’s health by implementing and enforcing regular health checks and awareness programs. Legal requirements for routine health checks and the provision of nutritional support from pregnancy through childbirth should be established to ensure that women receive the necessary care. Addressing malnutrition is especially critical in rural areas, where it is more prevalent. By enhancing their understanding of nutritional needs and resources, rural women will be better equipped to ensure the health and well-being of their families. A multi-faceted approach involving education, healthcare access, and nutritional support is crucial for advancing women’s empowerment and improving child health. Targeted policies and programs that address these areas will contribute to better health outcomes and greater empowerment for women, ultimately benefiting entire communities.

## Figures and Tables

**Table 1:** Countries selected within developing regions (N = 10).

Regions	Economies Developing	15–49 Age Women (Ever Married)	Years
Asia (Western)	Azerbaijan	8444	2005–2006
	Jordan	11,352	2011–2012
	Armenia	6116	2015–2016
Asia (Central)	Tajikistan	9656	2011–2012
	Kyrgyz republic	8208	2011–2012
Asia (Southern and Southeastern)	Pakistan	13,558	2012–2013
	Timor-Leste	13,137	2009–2010
	Cambodia	17,578	2013–2014
	India	124,385	2005–2006
	Nepal	12,674	2010–2011

Source: DHS.

**Table 2:** Ranking of countries according to CMEI in selected developing countries of Asia.

Regions	Countries	Less Empowered	Mediate Empowered	High Empowered	CMEI Mean Score	Rank
West Asia	Azerbaijan	19.5	70.1	10.4	3.1037	5
	Jordan	1.6	82.2	16.1	3.1377	4
	Armenia	2.1	81.2	16.7	3.2171	3
Central Asia	Tajikistan	25.5	64.2	10.3	2.8369	7
	Kyrgyz Republic	7.2	72.4	20.4	3.0061	6
South and Southeast Asia	Pakistan	30	66	4	2.7714	9
	Timor-Leste	17.2	79.1	3.6	2.7316	10
	Cambodia	2.4	68.3	29.4	3.2389	2
	India	14	79	7	2.8285	8
	Nepal	0.2	61	38.8	3.3673	1

**Table 3:** Empowerment of mothers as determinants of children in South and Southeast Asia.

	CIAF <-2SD		Height for Age <-2SD		Weight for Height <-2SD		Weight for Age <-2SD	
Independent Variables	Odds Ratio	Std. Err.	Odds Ratio	Std. Err.	Odds Ratio	Std. Err.	Odds Ratio	Std. Err.
Composite Maternal Empowerment Index [CMEI]								
Composite Maternal Empowerment Index	0.782 ***	0.010	0.933 ***	0.011	1.003	0.011	1.02 **	0.014
Mother’s BMI [REF: below 18.5 kg/m^2^]								
Greater than 18.5 kg/m^2^	0.921 ***	0.030	0.666 ***	0.017	0.603 ***	0.015	0.631 ***	0.018
Education of Mother [REF: No Education]								
5th class	1.48 ***	0.060	0.945	0.033	0.863 ***	0.029	0.885 ***	0.036
10th class	1.09 ***	0.047	0.791 ***	0.028	0.806 ***	0.027	0.881 ***	0.035
Above	0.716 ***	0.067	0.498 ***	0.039	0.554 ***	0.039	0.867 *	0.070
Age of Mother								
Age of Mother	1.06 ***	0.004	1.03 ***	0.004	1.01 **	0.04	0.965 ***	0.005
Maternal Age at her First Birth								
Age of Mother at her first birth MAFB	0.991	0.005	0.965 ***	0.004	0.977 ***	0.004	1.03 ***	0.006
Education of Father [REF: No Education]								
5th class	1.19 ***	0.051	1.01	0.038	0.963	0.035	0.906 **	0.039
10th class	0.782 ***	0.033	0.872 ***	0.031	0.906 ***	0.030	0.925 *	0.037
Above	0.598 ***	0.043	0.681 ***	0.041	0.778 ***	0.043	0.860 **	0.057
Age of Father								
Age	1.001 **	0.002	0.999 **	0.002	0.998	0.002	0.999	0.002
Household Head Gender [REF: Female]								
Male	0.984	0.048	0.885 ***	0.037	0.873 ***	0.034	1.003	0.046
Household Head Age								
Age of Household Head	0.994 ***	0.001	0.997 **	0.001	0.998	0.009	1.001	0.0011
Wealth Status [REF: Lowest Income or Poorest]								
Lowest Income	0.903 ***	0.041	0.845 ***	0.033	0.809 ***	0.030	0.826 ***	0.036
Middle income	0.787 ***	0.037	0.685 ***	0.027	0.671 ***	0.025	0.771 ***	0.035
Richer or Better-off	0.790 ***	0.041	0.603 ***	0.027	0.579 ***	0.024	0.690 ***	0.035
Highest Income	0.869 ***	0.054	0.501 ***	0.027	0.441 ***	0.022	0.604 ***	0.037
Total Number of Members of Household [REF: >5]								
>=5	1.02 ***	0.006	1.01 *	0.005	1.01	0.005	0.998	0.006
Location [REF: Rural areas]								
Urban Areas	0.850 ***	0.030	0.991	0.030	1.05 *	0.030	1.06 *	0.088
Ever Born Number of Children [REF less than 4]								
>=4	1.02 **	0.012	1.01	0.011	1.02 **	0.011	1.09 ***	0.015
Constant	0.144 ***	0.018	1.46 ***	0.163	2.002 ***	0.212	0.472 ***	0.059
Pseudo R^2^	0.081		0.065		0.055		0.023	
[Observations]	[33,574]		[32,666]		[35,262]		[35,268]	

CIAF, Composite Index of Anthropometric Failure. Level of significance < 0.01 = ***; level of significant < 0.005 = **; level of significance < 0.10 = *. REF [Reference Category] Source: [27].

**Table 4:** Empowerment of mothers as determinants of children in Central Asia.

	CIAF <-2SD		[Height for Age <-2SD]		[Weight for Height <-2SD]		[Weight for Age <-2SD]	
Independent Variables	Odds Ratio	Std. Err.	Odds Ratio	Std. Err	Odds Ratio	Std. Err.	Odds Ratio	Std. Err.
Mother/Women Empowerment								
Combined Maternal Empowerment Index [CMEI]	1.07	0.053	1.02	0.037	0.944	0.052	0.979	0.058
Mother’s BMI [REF: fewer than 18.5 kg/m^2^]								
Greater than 18.5 kg/m^2^	1.39 ***	0.349	0.963	0.158	0.862	0.215	0.850	0.224
Education of Mother [REF: No Education]								
5th class	Omit		0.690	0.392	0.923	0.787	1.59	1.79
10th class	0.356	4.56	0.586	0.90	0.728	0.552	1.30	1.35
Above	0.526	0.686	0.559	0.80	0.452	0.348	0.976	1.02
Age of Mother								
Age	0.965 **	0.014	0.97 **	0.010	0.985	0.017	1.02	0.019
MAFB								
Age of mother at first birth [MAFB]	1.02	0.017	1.03 **	0.013	1.001	0.021	0.986	0.022
Education of Father [REF: No Education]								
5th class	0.064 *	0.099	0.782	0.702	598	7.08	0.177	0.194
10th class	0.285	0.325	0.974	0.791	110	1.31	0.245 *	0.203
Above	0.246	0.281	0.963	0.783	113	1.34	0.266	0.221
Age of Father								
Age	1.02 **	0.012	1.01 *	0.009	1.004	0.014	0.986	0.022
Household Head Gender [REF: Female]								
Man	1.26 *	0.169	1.04	0.101	1.08	0.59	1.25	0.188
Head of Household								
Age of Head	0.995	0.003	0.997	0.002	1.01 *	0.004	1.01	0.004
Wealth status [REF: Poorest]								
Lowest Income	0.823	0.106	0.886	0.101	0.896	0.171	1.03	0.221
Middle income	0.685 ***	0.089	0.988	0.108	0.828	0.156	0.823	0.179
Better-off or Richer	0.506 ***	0.076	0.923	0.108	1.35 *	0.248	1.76 ***	0.352
Highest income	0.114 ***	0.027	1.03	0.137	1.81 ***	0.373	2.25 ***	0.500
Household Members [REF: Less than 5]								
>=5	0.884 ***	0.024	1.02	0.017	1.02	0.026	1.04	0.028
Location [REF: Rural areas]								
Urban Areas	0.885	0.129	0.966	0.097	0.905	0.138	0.867	0.138
Number of Children Ever Born [REF: Less than 4]								
>=4	1.037	0.047	1.04	0.034	1.13 ***	0.055	1.03	0.054
Constant	1.69	0.002	0.290	0.284	5.74	0.000	0.114	0.159
Pseudo R^2^	0.075		0.0024		0.023		0.024	
[Observation]	[5287]		[5355]		[5355]		[5355]	

CIAF, Composite Index of Anthropometric Failure. Level of significance < 0.01 = ***; level of significant < 0.005 = **; level of significance < 0.10 = * REF: [ Reference Category] Source: [27].

**Table 5:** Empowerment of mothers as determinants of children in West Asia.

	[CIAF <-2SD]		[Height for Age <-2SD]		[Weight for Height <-2SD]		[Weight for Age <-2SD]	
Variables (Independent)	Odds Ratio	Std. Err.	Odds Ratio	Std. Err.	Odds Ratio	Std. Err.	Odds Ratio	Std. Err.
Mother/Women Empowerment								
CMEI	0.959	0.104	0.989	0.021	1.001	0.036	1.024	0.030
Mother’s BMI [REF: less than 18.5 kg/m^2^]								
Greater to 18.5 kg/m^2^	Omitt		1.87	0.743	0.793	0.369	1.34	0.696
Education of Mother [REF: No Education]								
5th class	1.29	0.581	1.07	0.096	1.03	0.154	0.811 *	0.101
10th class	1.46	0.522	1.03	0.072	1.01	0.119	0.751 ***	0.071
Above	0.793	0.380	0.942	0.088	0.802	0.127	0.626 ***	0.081
Age of Mother								
Mother’s Age	0.989	0.029	0.997	0.006	0.989	0.010	1.001	0.008
MAFB								
Mother Age at First Birth	1.01	0.032	0.994	0.06	0.994	0.011	0.998	0.009
Education of Father [REF: No Education]								
5th class	0.871	0.343	0.874	0.073	0.733 **	0.101	0.818 *	0.096
10th class	0.738	0.256	0.871 *	0.064	0.692 ***	0.082	0.941	0.096
Above	0.792	0.342	0.819 **	0.076	0.709 **	0.107	0.863	0.111
Age of Father								
Father’s Age	0.987	0.022	1.01 **	0.004	1.00	0.007	0.996	0.006
Household Head [REF: Female]								
Man	1.47	0.651	1.01	0.114	0.843	0.165	0.719 *	0.127
Head of Household								
(Age)	[1.01]	0.012	1.001	0.002	0.999	0.004	0.999	0.004
Wealth Status [REF: Poorest]								
Lowest Income	2.009 **	0.730	0.917	0.068	1.01	0.126	0.891	0.091
Middle income	0.763	0.326	1.10	0.080	0.936	0.118	0.695 ***	0.075
Better-off	1.41	0.553	1.28 ***	0.098	1.12	0.145	1.11	0.119
Highest Income	1.81	0.750	1.38 ***	0.119	1.51 ***	0.210	1.79 ***	0.029
Household Members [REF: Less than 5]								
>=5	0.942	0.072	1.001	0.015	1.05 **	0.024	1.01	0.021
Location [REF: Rural areas]								
Urban Areas	1.12	0.299	0.681 ***	0.037	1.04	0.097	0.857 *	0.069
Number of Children Ever Born [ REF: Less than 4]								
>=4	1.011	0.112	0.912 ***	0.020	0.941 *	0.034	0.977	0.029
Constant	0.038 ***	0.037	0.125 ***	0.056	1.40 ***	0.082	0.135 ***	0.081
Pseudo R^2^	0.017		0.0007		0.0085		0.0124	
[Observations]	[18,118]		[18,201]		[18,208]		[18,201]	

CIAF, Composite Index of Anthropometric Failure. Level of significance < 0.01 = ***; level of significant < 0.005 = **; level of significance < 0.10 = *; standard error. REF [Reference Category] Source: [27].

**Figure A1:**
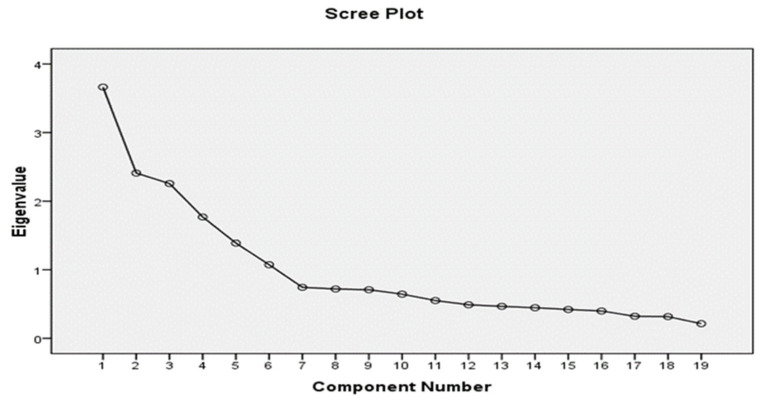
Scree plot of eigenvalues of factors of maternal empowerment.

## Data Availability

Data used in this manuscript are publicly available at the DHS website: https://dhsprogram.com/Data/, accessed on 12 March 2025.

## Abbreviations

BLR	Binary logistic regression
CIAF	Composite Index of Anthropometric Failure
CMEI	Composite maternal empowerment index
MAFB	Mother’s age at first birth
BMI	Body Mass Index

## Appendix A

**Table A1:** Operational definition of the variables and measurement scale.

[Variables]	[Description]	[Measurement Scale]
Dependent Variables		
[Child Malnutrition]	Stunting [height for age]	[1 = Stunting [z scores <−2 SD], 0 = Otherwise]
	Wasting [weight for height]	[1 = Wasting [z scores <−2 SD], 0 = Otherwise]
	Underweight [weight for age]	[1 = Underweight [z scores <−2 SD], 0 = Otherwise]
	[Composite Index of Anthropometric Failure]	[1 = Yes, 0 = No]
[Independent Variables]		
[Predisposing Factors]	[Father’s Education]	[0 = No Education 1 = Primary 2 = Secondary 3 = Higher]
	[Father’s Age]	[Discrete]
	[Mother’s Education]	[0 = No Education 1 = Primary 2 = Secondary 3 = Higher]
	[Gender of Head of Household]	[Male = 1, Female = 0]
	[Age of Head of Household]	[Continuous]
	[Total Children Ever Born]	[Discrete]
	[Total Number of Household Members]	[Discrete]
[Enabling Factors]	Composite Maternal Empowerment Index (CMEI)	[Continuous]
[Need-Based Factors]	Mother’s Body Mass Index (MBMI)	[0 = less than 18.5 kg/m^2^ 1 = more than 18.5 kg/m^2^]
	[Mother’s Age at First Birth]	[0 = Less than 20 Years 1 = 20–25 2 = Above than 30]
[Environment]	[Locality]	[1 = Urban, 0 = Rural]
	[Household Wealth Index]	[0 = Poorer 1 = Middle 2 = Richer 3 = Richest]

Note: SD, Standard Deviation.

**Table A2:** The measurement scale of the dimensions of maternal empowerment.

[Dimensions]	[Description]	[Measurement Scale for DHS Data]
[Work Status (WS)]	[Respondent is currently working]	[No = 0, Yes = 1]
	[Respondent’s employment status]	[0 = did not work, 1 = unskilled manual 2 = Skilled manual, 3 = household domestic, 4 = agricultural − self-employed, 5 = clerical 6 = agricultural − employee, 7 = sales, 8 = services 9 = professional/technical/managerial]
[Awareness (AW)]	[Respondent watching TV]	[0 = not at all, 1 = almost daily, 2 = at least once a week]
	[Respondent reading newspaper/magazines]	[0 = not at all, 1 = almost daily, 2 = at least once a week]
	[Respondent listening to a radio]	[0 = not at all, 1 = almost daily, 2 = at least once a week]
	[Heard about family planning on radio]	[0 = not at all, 1 = less than once a week 2 = at least once a week, 3 = almost daily]
	[Heard about family planning on TV]	[0 = not at all, 1= less than once a week 2 = at least once a week, 3 = almost daily]
	[Heard about family planning from newspapers]	[0 = not at all, 1 = less than once a week 2 = at least once a week, 3 = almost daily]
[Decision-Making (DM)]	[The decision to spend women’s husband’s earnings]	[0 = Husband has no earnings, 1 = Someone else, 2 = Husband/partner alone, 3 = Respondent and husband/partner 4 = Respondent alone]
	[A decision on women’s health]	[1 = Someone else, 2 = Husband/partner alone 3 = Respondent and husband/partner, 4 = Respondent alone]
	[Decision about large household purchases]	[1 = Someone else, 2 = Husband/partner alone 3 = Respondent and husband/partner, 4 = Respondent alone]
	[Decision about visits to family or relatives]	1 = Someone else, 2 = Husband/partner alone 3 = Respondent and husband/partner, 4 = Respondent alone]
[Self Esteem (SE)]	[Beating justified if wife argues with husband]	[0 = Yes justified, 1 = Not justified]
	[Beating justified if wife neglects children]	[0 = Yes justified, 1 = Not justified]
	[Beating justified if without telling the husband]	[0 = Yes justified, 1 = Not justified]
	[Beating justified if the wife refuses to have sex with the husband]	[0 = Yes justified, 1 = Not justified]
	[Beating justified if a wife burns food]	[0 = Yes justified, 1 = Not justified]
[Self-Confidence (SC)]	[Obtaining medical help for self: want to go alone]	[0 = Big problem, 1 = Not a big problem]
	[Getting medical help for self: obtaining money for treatment]	[0 = Big problem, 1 = Not a big problem]

Source: Demographic and Health Survey (DHS-V 2003–2008, DHS-VI 2008–2013, DHS-7 2013–2018).

**Table A3:** KMO measure of sampling adequacy and Bartlett’s test of sphericity.

KMO and Bartlett’s Test		
Kaiser–Meyer–Olkin Measure of Sampling Adequacy.		0.736
Bartlett’s Test of Sphericity	Approx. Chi-Square	2,138,734
	df	171
	Sig.	0.00

**Table A4:** Findings of PCA: The Varimax Rotated Factor Matrix.

Sr.	Variables	Work Status	Awareness	Decision Making	Self Esteem	Self Confidence	Commulities
1	The beating is justified if wife argues with husband				0.814		0.674
2	The beating is justified if the wife neglects the children				0.827		0.690
3	The beating is justified if wife goes outside without telling husband				0.817		0.682
4	The beating is justified if wife refuses to have sex with husband				0.713		0.535
5	The beating is justified if wife burns food				0.741		0.557
6	The respondent currently working	0.919					0.855
7	The respondent employment status	0.909					0.833
8	Respondent read newspaper or magazine		0.664				0.488
9	The respondent listen radio		0.526				0.402
10	The respondent watching TV.		0.653				0.504
11	Heard about family planning on radio last few.		0.581				0.432
12	Heard about family planning from TV.		0.734				0.582
13	Heard family planning in newspaper		0.711				0.521
14	The decision to spend women’s husband earnings.			0.668			0.464
15	The decision to spend women’s health care			0.751			0.576
16	The decision about household large purchases			0.811			0.663
17	The decided to their relatives			0.755			0.586
18	Taking medical help for self: not want to go alone					0.833	0.724
19	Medical help for self: getting money for treatment					0.844	
